# Recurrent broad ligament leiomyosarcoma with pancreatic and thigh metastasis: a case report

**DOI:** 10.1186/s12893-020-00804-w

**Published:** 2020-06-29

**Authors:** Xuan Tian, Xin Yan, Jun Wu, Hongli Song, Zhongyang Shen

**Affiliations:** 1 School of Medicine, Nankai University, Tianjin, 300071 China; 2 The Second Hospital of Jilin University, Changchun, 130033 China; 3 Department of Organ Transplantation, Tianjin First Central Hospital, Tianjin, 300192 China

**Keywords:** Pancreas, Thigh, Metastases, Broad ligament leiomyosarcoma

## Abstract

**Background:**

Leiomyosarcoma (LMS) is an uncommon mesenchymal neoplasm, which infrequently metastasizes to pancreas and thigh. Clinical presentation and imaging findings of metastatic broad ligament LMS are often nonspecific. Complete excision plays an important role in treatment of patients with localized LMS.

**Case presentation:**

Here, we report a case of a 33-year-old woman with recurrent broad ligament LMS metastasizing to pancreas and thigh. Previously, she was diagnosed with broad ligament LMS and underwent hysterectomy, bilateral salpingo-oophorectomy. The disease-free interval was 2.5 years until metastases were found. Computerized tomography (CT) of abdomen and thighs, magnetic resonance imaging (MRI) of thighs and whole-body 18-fluorodeoxyglucose positron emission tomography - computed tomography (PET-CT) performed, revealed pancreatic and thigh metastasis. Ultrasonography-guided biopsy and histological examinations confirmed LMS at both the sites. Pancreatic metastasis was completely resected first. Then the patient underwent surgical resection of thigh metastasis when both chemotherapy and radiotherapy failed. She recovered well and remained free of disease recurrence in the 2 years follow-up.

**Conclusions:**

Though imaging lacks specificity, it is a valuable asset in assessing the burden of disease and characterizing lesions while histological examination with immunohistochemistry is helpful for the diagnosis of LMS. Complete surgical resection of all metastatic sites where-ever feasible should be strongly considered in a treated case of broad ligament LMS with a durable disease-free interval.

## Background

Leiomyosarcoma (LMS) is a subtype of soft tissue sarcoma. It is a very uncommon mesenchymal neoplasm with an incidence < 1/100000/year [1]. LMS usually arises from uterus, retroperitoneum, abdomen, large blood vessels, extremities and other soft tissues [2, 3]. Molecular mechanisms underlying its pathogenesis are not clear and early diagnosis and treatment are difficult. Management of LMS requires a multidisciplinary team [4]. Surgical resection is the cornerstone treatment of patients with localized LMS [5]. Radiotherapy and chemotherapy may not show benefit in all cases. Patients with metastases have a dismal prognosis, and the common metastatic sites of LMS are lung and liver [5]. It infrequently metastasizes to pancreas and thigh. After taking symptomatology, co-morbidities, disease-free interval and morbidity of surgery into account, surgical resection of all resectable metastases should be strongly considered where ever feasible [3].

We encountered a case of recurrent LMS of broad ligament of uterus with pancreatic and right thigh metastasis which were successfully treated by surgery. We report this case and discuss the characteristics, diagnosis and management of it, hoping it could provide some hints and inspirations for diagnosis and treatment of metastatic LMS.

## Case presentation

A 33-year-old woman with no family history of cancer presented to a local hospital in November 2014 with a palpable mass in hypogastric region. Transvaginal ultrasonography showed a 12 × 8 cm irregular hypoechoic mass with spot blood flow signals and clear boundary on left rear part of the uterus. She was diagnosed with uterine leiomyoma, and underwent an open myomectomy. Pathologic examination revealed a broad ligament LMS, and immunohistochemical analyses showed positivity for SMA and a Ki 67 level of 30%. Subsequently hysterectomy, bilateral salpingo-oophorectomy and omentectomy were performed. No tumor cells were detected in sections taken from tubes, ovaries and omentum. She recovered well and then discharged from the hospital.

**Fig. 1:**
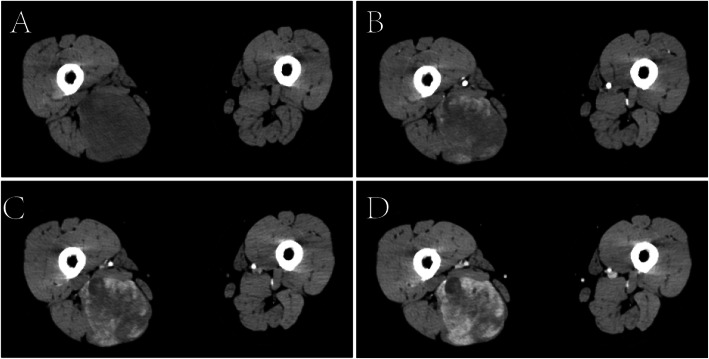
CT scan of right thigh showing a low-density mass with delayed enhancement. **a** plain scan, **b** arterial phase, **c** venous phase, **d** delayed phase

**Fig. 2:**
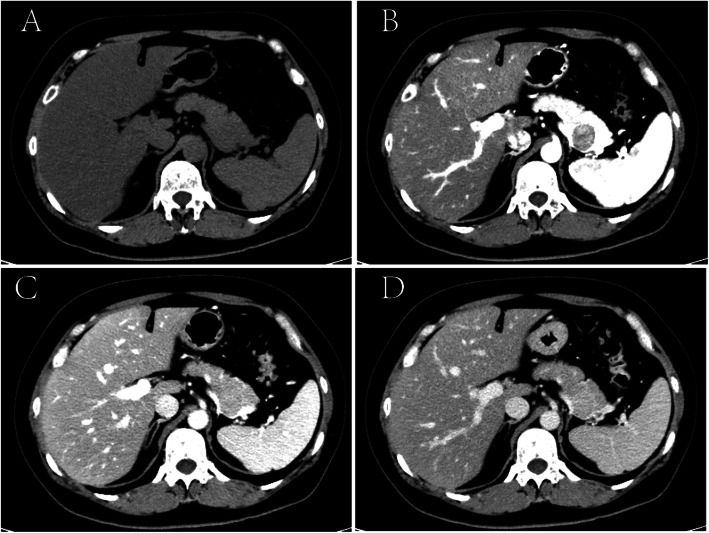
CT scan of abdomen showing a round low-density mass in the tail of pancreas with heterogeneous enhancement. **a** plain scan, **b** arterial phase, **c** venous phase, **d** delayed phase

In November 2017, she was referred to our hospital for a mass in the right thigh. On physical examination, a firm mass measuring 10 cm in diameter with tenderness was felt on the back of right thigh, and there were not any positive abdominal findings. Laboratory findings including serum amylase, serum lipase and serum tumor markers (CA19–9 and CEA) were all within normal reference ranges. CT scan of thighs showed a low-density mass with delayed enhancement in the back of right thigh (Fig. 1), and MRI of thighs showed long T1 and long T2 signals on plain scan and high signals on fat suppression imaging. A diagnosis of LMS was established on ultrasonography-guided core needle biopsy, and immunohistochemical study showed positive staining for SMA, Vimentin, Desmin and H-Caldesmon, and negative staining for S-100, CD117, DOG-1 and CD34. To evaluate the overall condition of the patient, a whole-body 18-fluorodeoxyglucose positron emission tomography-computed tomography (PET-CT) was performed, which revealed one hypermetabolic lesion on the right thigh and another on the tail of pancreas. Abdominal CT scan showed a 22 mm * 23 mm rounded low-density mass on the tail of pancreas with heterogeneous enhancement in arterial phase (Fig. 2).

**Fig. 3:**
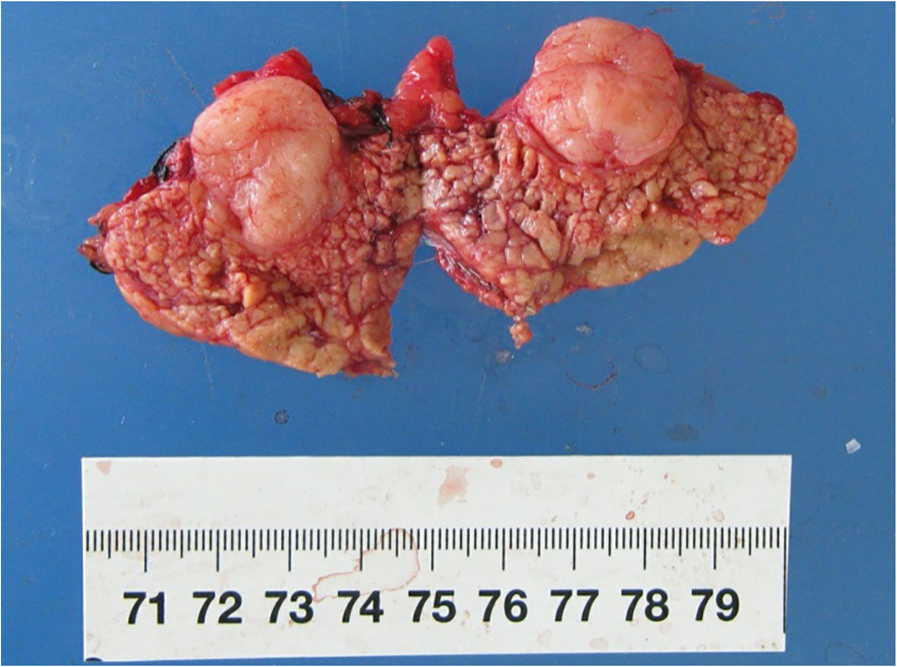
Gross pathology of distal pancreatectomy specimen with cut-section showing tumor as whitish fish-liking mass with complete capsule

**Fig. 4:**
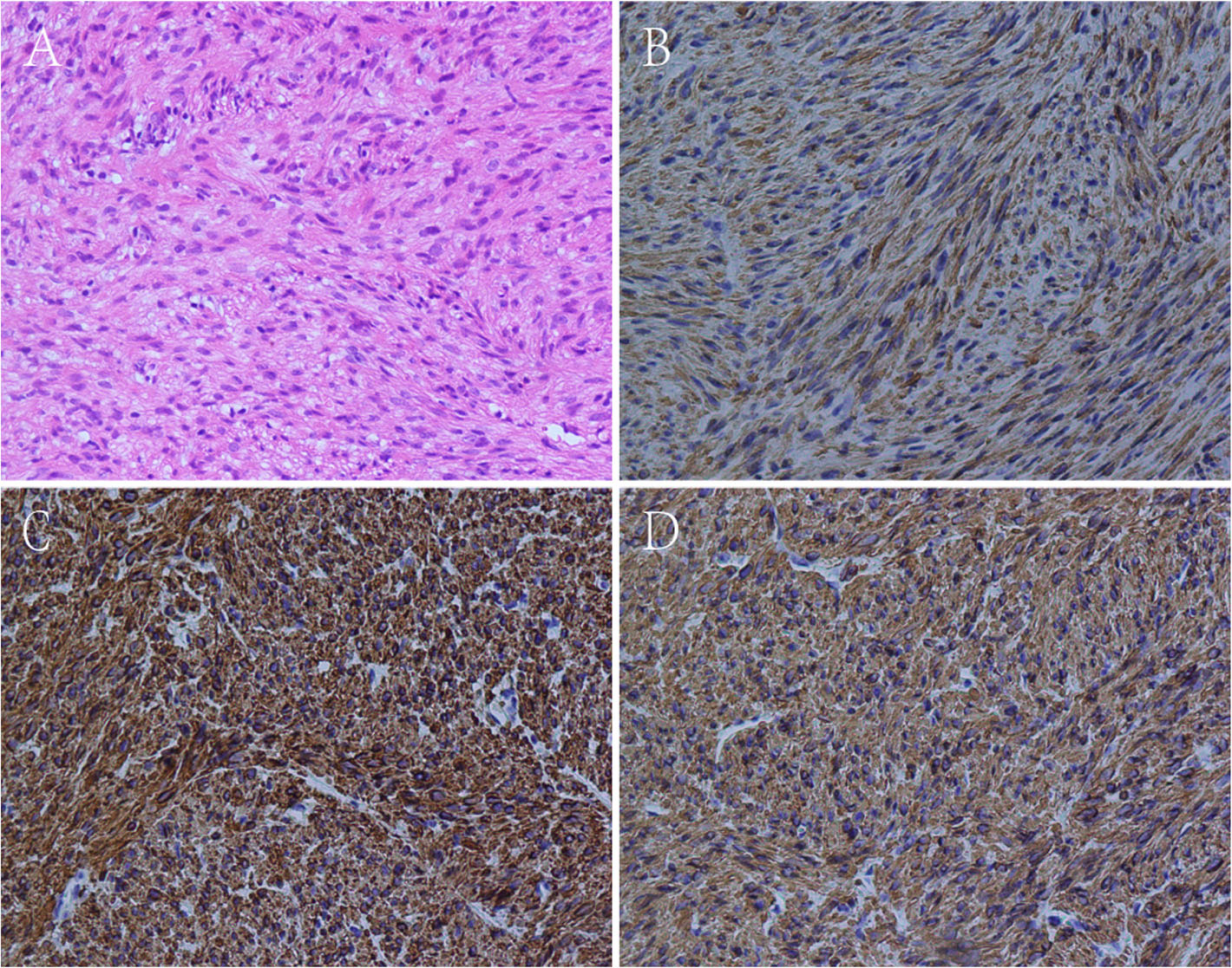
Histological and immunohistological features of metastatic LMS lesion of pancreas. **a** hematoxylin and eosin staining showed that cells were spindle, with necrosis, mild atypia, and mitotic figures **b-d** Immunohistochemistry revealed smooth muscle differentiation. Just like most LMSs, tumor cells showed positive staining for Desmin, H-Caldesmon and SMA. B Desmin, C H-Caldesmon, D SMA

A group of experts including surgeons, oncologists, radiologists and pathologists met to provide Multi-Disciplinary Treatments. The surgical team included experienced general and orthopaedic surgeons. Resectability of the two leisons was evaluated by the surgical team of the multidisciplinary team. Neoadjuvant therapy was administered as the tumor was deemed to be borderline resectable with concerns to its proximity to neurovascular bundle. Therefore, we first performed a distal pancreatectomy. The cross-section of the en bloc resected tumor was a whitish fish-liking mass with complete capsule (Fig. 3). The frozen pathological examination supported the diagnosis of an LMS with necrosis, mild atypia, and 17-18 mitosis per 10 high power fields (HPF) (Fig. 4). Immunohistochemical staining was positive for SMA, desmin, h-caldesmon, Ki67 (positive rate 30%), and negative for S-100, CD117, dog-1, CD34.The tumor in the thigh received external-beam radiotherapy (25Gy given in 10 fractions on week days) and chemotherapy (Lobaplatin 50 mg/m2 intravenously daily) for 3 weeks, to which the tumor showed no response. Therefore, the tumor was resected completely with a negative margin. Postoperative pathologic examination was consistent with the former ultrasonography-guided biopsy and frozen pathologic examination of pancreatic tumor. The patient was discharged 5 days after the operation, and free of disease recurrence in the 2 years follow-up.

## Discussion and conclusions

LMS metastasis to the pancreas is uncommon, and there are few reports. A literature review of LMS metastasis to the pancreas is presented in Table 1 [6–13].

**Table 1:** Literature review of LMS metastasis to the pancreas

Reference	Sex	Age	Primary site	Metastatic site	Interval	Treatment for metastasis	Survival
Alonso et al. [6]	F	35	Uterus	Pancreas, lung, gallbladder, liver	20 months	Surgery, chemotherapy	>18 months
Dima et al. [7]	F	67	Uterus	Pancreas, lung	19 months	Surgery, chemotherapy	>3 months
Hernandez et al. [8]	F	46	Uterus	Pancreas, lung	4 years and 2 months	Surgery, chemotherapy	>10 months
Iwamoto et al. [9]	F	46	Uterus	Pancreas, lung	2 years	Surgery, chemotherapy	>8 months
Koh et al. [10]	F	66	Thigh	Pancreas	NA, 9 months	Surgery	>9 months
Ogura et al. [11]	F	60	Thigh	Pancreas	NA	Surgery	>1 year
Ozturk et al. [12]	F	40	Uterus	Pancreas	4 years and 5 months	Surgery	>6 months
Sweeney et al. [13]	F	51	Uterus	Pancreas	1 year	Chemotherapy	>1 year
Present case	F	36	Broad ligament	Pancreas, thigh	3 years	Surgery, chemotherapy, radiotherapy	>2 years

*F* female, *Interval* interval between primary tumor and pancreatic metastasis, *Survival* survival after diagnosis of pancreatic metastasis, *NA* not available

A retrospective study of 13 patients with metastatic pancreatic LMS showed that the median interval between diagnosis of metastases and primary LMS was 24 moths (range, 1–77 moths), and all patients were associated with metastases to other organs [14]. In this case, pancreatic and thigh metastasis were found 3 years after resection of the broad ligament LMS, showing a long interval between the treatment of primary LMS and the appearance of pancreatic metastasis. When there is a pancreatic metastasis, clinicians should be alerted to metastases in other organs. Metastatic pancreatic LMS has no specificity in clinical and image findings, so it is difficult to make an exact diagnosis preoperatively. In contrast-enhanced CT scan, most of metastatic pancreatic LMSs are hypovascular [14], which need to be carefully differentiated from pancreatic ductal adenocarcinoma. In this case, the pancreatic lesion showed heterogeneous enhancement that was lower than the surrounding normal pancreas in arterial phase, and the same enhancement degree with the surrounding tissue in venous phase. Unlike primary pancreatic ductal adenocarcinoma, metastatic pancreatic LMS has a clear boundary and no manifestations such as dilation of pancreatic bile duct and atrophy of pancreas. These signs are helpful for differentiation. It is interesting to note that the lesion in the thigh showed delayed enhancement, which was different from the enhancement mode of the pancreatic lesion. PET-CT scan can detect and localize metastases. Given that metastatic pancreatic LMS is often accompanied by metastasis to other organs, PET-CT is necessary to estimate the burden of the tumor.

Core needle biopsy is of great value for diagnosis, although inadequate sampling may lead to false negative result. At present, pathological diagnosis of LMS mainly depends on cell morphology. LMS cells are significantly different from smooth muscle cells, characterized by spindle shaped cells arranged in clusters, with rich eosinophilic cytoplasm and hyperchromatic enlongated nucleus [5]. Most LMSs are positive for smooth muscle actin, desmin, and h-caldesmon [3, 5], which are advantageous to differential diagnosis.

Considering neither non-invasive imaging nor pathological examinations can lead to accurate and specific diagnosis of LMS, researchers are eager to find a diagnostic and therapeutic method at molecular level. A study applicating circulating miRNAs as uterine LMS biomarkers found that combination of mir-1246 and mir-191-5p reached a diagnostic rate of 97% [15]. Xiangqian Guo et al. [2] identified 3 LMS molecular subtypes associated with distinct clinical outcomes, making a good exploration of individualized and comprehensive therapy of LMS. Subtype I is most closely similar to smooth muscle differentiation and predicts a favorable outcome. In contrast, subtype II shows no significant smooth muscle differentiation and is associated with unfavourable prognosis. Subtype III shows a preference for uterus.

Some high-frequency gene mutations may act as oncogenes in tumorigenesis and progression of LMS, such as TP53, Rb, ATRX, med12, and PTEN et al. [16, 17]. Mutations of AKT1 (protein kinase b-alpha) and PTEN indicate that PI3K-Akt-mTOR signal pathway play a vital oncogenetic role in LMS [18]. It has been demonstrated that rapamycin, an inhibitor of mTOR, has a certain effect in LMS mice model [19]. In addition, Cantharidin and protease inhibitor (MG-132) also can effectively inhibit viability of LMS cells in vitro [20].

Treatment of metastatic LMS involves a multi-strategy in stepwise management, such as surgery, systemic treatment and radiotherapy [4]. Qian et al. [21] analyzed data of 239 patients with metastatic extremity LMS, and found that surgery plus chemotherapy improved survival of these patients. However, in this case, radiotherapy and chemotherapy all failed. Complete surgical resection was performed with R0 margin status considering the prolonged disease-free interval, feasible resectability of the two isolated lesions, young age and good surgical tolerability of the patient. Finally, clinical benefit with long disease-free survival was achieved by R0 resection (negative margins). It has already been proved that resection of metastatic soft tissue sarcomas in the lung and liver could bring survival benefits [22]. In accordance with NCCN Clinical Practice Guidelines in Oncology for soft tissue sarcoma (version 2, 2018, [23], complete surgical resection of isolated and resectable metastases should be considered in treatment of recurrent broad ligament LMS with a durable disease-free interval.

## Data Availability

All data generated or analysed during this study are included in this published article.

## Abbreviations

CA-199: Carbohydrate antigen 19–9
CEA: Carcino-embryonic antigen
CT: Computed tomography
LMS: Leiomyosarcoma
MRI: Magnetic resonance imaging
PET-CT: Positron emission tomography - computed tomography
SMA: Smooth muscle actin
